# A knowledge-based taxonomy of critical factors for adopting electronic health record systems by physicians: a systematic literature review

**DOI:** 10.1186/1472-6947-10-60

**Published:** 2010-10-15

**Authors:** Víctor H Castillo, Ana I Martínez-García, JRG Pulido

**Affiliations:** 1 Faculty of Mechanics and Electrical Engineering, University of Colima, México; 2 Department of Computer Science, CICESE, Ensenada B.C., México; 3 Faculty of Telematics, University of Colima, México

## Abstract

**Background:**

The health care sector is an area of social and economic interest in several countries; therefore, there have been lots of efforts in the use of electronic health records. Nevertheless, there is evidence suggesting that these systems have not been adopted as it was expected, and although there are some proposals to support their adoption, the proposed support is not by means of information and communication technology which can provide automatic tools of support. The aim of this study is to identify the critical adoption factors for electronic health records by physicians and to use them as a guide to support their adoption process automatically.

**Methods:**

This paper presents, based on the PRISMA statement, a systematic literature review in electronic databases with adoption studies of electronic health records published in English. Software applications that manage and process the data in the electronic health record have been considered, i.e.: computerized physician prescription, electronic medical records, and electronic capture of clinical data. Our review was conducted with the purpose of obtaining a taxonomy of the physicians main barriers for adopting electronic health records, that can be addressed by means of information and communication technology; in particular with the information technology roles of the knowledge management processes. Which take us to the question that we want to address in this work: "What are the critical adoption factors of electronic health records that can be supported by information and communication technology?". Reports from eight databases covering electronic health records adoption studies in the medical domain, in particular those focused on physicians, were analyzed.

**Results:**

The review identifies two main issues: 1) a knowledge-based classification of critical factors for adopting electronic health records by physicians; and 2) the definition of a base for the design of a conceptual framework for supporting the design of knowledge-based systems, to assist the adoption process of electronic health records in an automatic fashion. From our review, six critical adoption factors have been identified: user attitude towards information systems, workflow impact, interoperability, technical support, communication among users, and expert support. The main limitation of the taxonomy is the different impact of the adoption factors of electronic health records reported by some studies depending on the type of practice, setting, or attention level; however, these features are a determinant aspect with regard to the adoption rate for the latter rather than the presence of a specific critical adoption factor.

**Conclusions:**

The critical adoption factors established here provide a sound theoretical basis for research to understand, support, and facilitate the adoption of electronic health records to physicians in benefit of patients.

## Background

In this study, the concept of electronic health records is used to describe applications for manipulating and processing any information, of an individual, that resides in electronic systems for the purpose of providing health care and health related services [1]. Based on this, some examples of information systems that might have an impact on the improvement of health services and that can be considered as electronic health records are: computerized physician prescription, computerized physician order entry, electronic medical records, electronic alerts, automated decision support, and electronic capture of clinical data that enables service quality improvement. Physicians have a central role in the use of electronic health records, as they are who provide much of the information that the systems handle in their automated processes.

These systems have been widely diffused in the medical domain, not only for the reduction in costs of information and communication technology, but also for supporting physicians tasks in three directions: 1) reducing medical error rates [2,3]; 2) supporting decision making activities [2,4]; and 3) incrementing cost-benefit ratio and improving the quality of health services [2,5,6]. Nevertheless, it has been reported that low adoption levels of electronic health records exist [2,7,8,87]. Based on Rogers [9], the term adoption refers to making the decision to make full use of an innovation (in this case electronic health records) as the best course of action available, in this paper we will use the term in that sense. The problem with the adoption of electronic health records is dramatic, for example, for 2010 it has been projected for the USA a value close to 50% in their adoption rate [2,10]. Such rate might negatively affect the quality and cost of the health services in this country.

Adoption studies can be grouped in a more general theory called diffusion of innovations, as proposed by Rogers [9], which helps to explore and explain why some new technologies spread faster and wider than others. In this theory, an innovation is an idea, practice, or object that is perceived as new by an adoption unit (e.g. an individual or an organization). The existing literature on adoption studies is mainly oriented towards two aspects: 1) the identification of the perceptions and attitudes towards information and communication technology, and 2) the identification of the characteristics of technology and its adopters. From our literature review, we found very few proposals [11] intended to support and promote, in an automatic manner by means of proactive technology, the adoption of electronic health records; furthermore, some of the proposed solutions were not related to the medical domain [12]. Since we want to provide automated means to support and promote the adoption of electronic health records in the latter domain, the last aspect is very important because the medical domain has a very particular organizational culture concerning the use of information systems; for instance, the habit of physicians continuously accessing information, or the way in which they request assistance to solve problems related to the use of technology. Like in other innovations, the adoption-decision process of electronic health records is based on knowledge; therefore in this paper we propose a knowledge-based taxonomy of the critical factors for adopting these systems. The proposed taxonomy is intended to be a guide for supporting the adoption process of the electronic health records with the assistance of information and communication technology. Our review differs from previous studies [13-19] regarding the following elements:

• Domain: our main interest is the medical domain; the taxonomy is focused on this and takes into account its culture in the use of information systems. Some studies facilitate the understanding of successful adoption and the use of information systems, but such studies are not focused on the adoption of electronic health records [13,14].

• Approach: our taxonomy is aimed towards a knowledge-based technology approach. Some studies propose critical factors for adopting electronic health records, but they do not recommend the elements for supporting their adoption by automatic means [15,18].

• Study subjects: our taxonomy does address physician personnel. However, some work is oriented toward the medical domain, and is not focused on physicians, i.e. it is aimed at patients [16], nurses [19], or nations [17].

**Figure 1:**
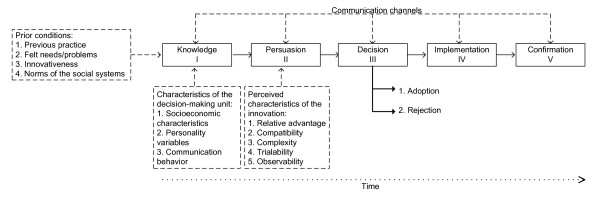
**The innovation-diffusion process proposed by Rogers **[9].

The innovation-decision process, showed in Figure 1, is composed of five stages [9]: the first stage is called knowledge, in which an individual, or a different adoption unit, has the first knowledge of an innovation; in the second stage, also known as persuasion, an individual forms an attitude towards the innovation; the third stage is called decision, this is critical because in this stage an individual decides, based on knowledge acquired in previous stages, to adopt or reject an innovation; in the fourth stage, implementation, an adoption unit implements an innovation; finally, in the stage of confirmation, an individual ratifies his decision to adopt or reject an innovation. As Rogers states [9], knowledge is a critical element in the innovation-decision process, this process is an information-seeking and information-processing activity in which an adoption unit is motivated to reduce uncertainty about the advantages and disadvantages of an innovation.

On the other hand, technology can be used for creating, storing, distributing, and applying knowledge. Each one of these knowledge processes can be implemented with information and communication technology roles [20] such as those presented in table 1; e.g., data mining can be used for creating knowledge, whereas electronic bulletin boards can be utilized for knowledge distribution. Therefore, technology is an instrument that could be used to reduce uncertainty about an innovation [9].

**Table 1:** Information and communication technology roles in the knowledge management processes

Knowledge management process	Information and communication technology roles
Knowledge creation	Data mining, learning tools, combination of new products of knowledge.

Knowledge storage	Electronic bulletin boards, knowledge repositories, database, support to individual and organizational memory, access to intergroup knowledge.

Knowledge distribution	Electronic bulletin boards, discussion forums, knowledge directories, more extended internal networks, availability of more communication channels, fast access to knowledge sources.

Knowledge application	Expert systems, workflow systems, knowledge applied in multiple locations, fast application of new knowledge using automatic workflow.

**Figure 2:**
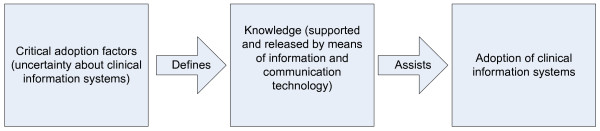
**Using knowledge for supporting adoption of clinical information systems**.

According to all the latter, the aim of this review is to provide a taxonomy to identify the critical adoption factors of electronic health records, that can be supported by information and communication technology, in a proactive form, to assist the users of these systems during their adoption process. This taxonomy is based on a list of critical adoption factors of electronic health records, classified from a knowledge point of view (Figure 2); where, as mentioned earlier, knowledge is an important element for decreasing the uncertainty towards them.

**Figure 3:**
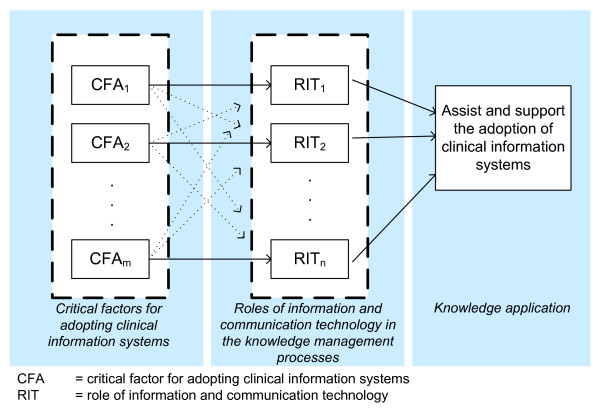
**Use of the critical adoption factors of clinical information systems to inform the development of systems for supporting its adoption process**.

In this way, every critical adoption factor could be associated with one or several of the information and communication technology roles of the knowledge management processes (creation, storage, and/or distribution) to apply such knowledge for assisting the adoption of the electronic health records (Figure 3).

## Methods

**Figure 4:**
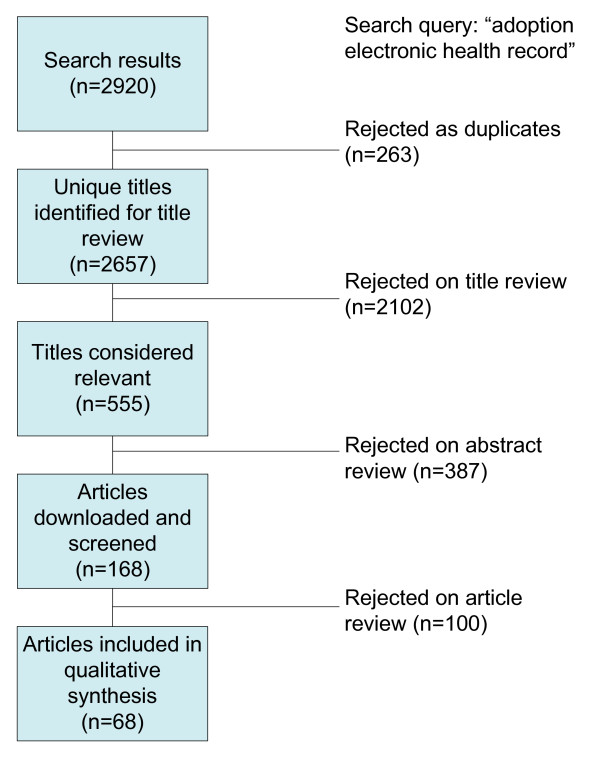
**Flow diagram of included and excluded studies**.

We have searched for relevant English language papers based on keywords within their title and abstract. Since knowledge helps to reduce uncertainty about an innovation, and technology can provide that knowledge timely and automatically, our review was conducted to answer the question "What are the critical adoption factors of electronic health records that can be supported by information and communication technology?" and was based on the PRISMA statement [21]. Figure 4 shows the search strategy used to identify relevant articles, and this is explained next.

### Study Identification and eligibility

First, some criteria for inclusion and exclusion of studies were established. We included studies that met the following two criteria: they were published between January 1, 1985, and December 31, 2009; and they describe critical adoption factors for adopting electronic health records by physicians, e.g. if they focused specifically on critical factors for nurse and/or administrative personnel, the studies were excluded. Although it is important to analyze the adoption of electronic health records by other stakeholders in the medical domain, as we stated before, physicians have a significant position in the use of these systems. We then excluded studies if they described critical adoption factors which can not be supported by the information and communication technology roles of the knowledge management processes, e.g. the establishment of financial incentives for the organizations that invest on technology has been defined as a critical adoption factor of electronic medical record systems [22], however, the promotion of such financial incentives using technology is slightly feasible. Critical adoption factor studies presented as posters or editorials were also excluded.

### Information sources and search strategy

We searched for journals and proceedings studies about information and communication technology as support of medical tasks in the following electronic libraries: IEEE, ACM, PubMed Central, Springer Link, Wiley InterScience, ScienceDirect, SAGE, and Scirus. The selected electronic libraries take into account that chosen journals and proceedings included articles with proposals for using information and communication technology to support automatic processes in the medical domain. To avoid excluding relevant articles for our review, we considered results from the Scirus search engine, which has a broad scientific spectrum and a coarser granularity in search in the scientific area, but includes useful results for purposes of our review. As mentioned earlier, we searched in selected journals and proceedings for articles published between January 1, 1985, and December 31, 2009. In the IEEE and ACM search engines, we searched for the keyword *adoption electronic health record *in document titles and abstracts. In addition we selected periodicals and conference proceedings publications. In the PubMed Central search engine, we also searched for the keyword *adoption electronic health record *in title and in citation+abstract. In the Springer Link search engine, first we searched for *adoption electronic health record *in all items and then we excluded books, series, and web content results. In the ScienceDirect search engine, we searched for the keyword *adoption electronic health record *in the documents' title and abstract. In the SAGE search engine, we searched for the keyword *adoption electronic health record *in the paper title and in full text/abstracts for the selected journals. Finally, in the Scirus search engine, we searched for the keyword *adoption electronic health record *in the complete documents for articles and scientist homepages. The search keyword is very wide, and provides a large number of studies that do not correspond to the objective of the literature review, however, it also provides the possibility to review work that meets the two criteria that concerns us: 1) addressing the issue of innovation adoption in medical field, and 2) including innovations concerning electronic health records.

### Review criteria, data extraction, and study selection

Two reviewers independently assessed each article for eligibility criteria. A third reviewer reviewed by adjudication, in cases of disagreement. While reviewing each study, the same two reviewers used standardized forms to independently extract and record four aspects: study id, list of critical adoption factors, country where the study was accomplished, type of practice, study setting, a knowledge-based classification in which critical adoption factors might engage type of electronic health records use, and study type. To collect the critical adoption factors for adopting electronic health records, the reviewers examined the critical adoption factors, defined in each study, taking into account the information and communication technology roles of the knowledge management processes presented in table 1. From the selected papers, the reviewers then compared the classification. Discrepancies between these two reviewers were resolved by a third reviewer.

## Results

Searching the online databases resulted in 2,920 articles from IEEE, ACM, PubMed Central, Springer Link, Wiley InterScience, ScienceDirect, SAGE, and Scirus. From the 2,581 titles that were initially identified, 441 were from IEEE, 279 from ACM, 971 from PubMed Central, 542 from Springer Link, 42 from Wiley InserScience, 28 from ScienceDirect, 278 from SAGE and 339 from Scirus. After removing duplicates and going through a screening process, we identified 168 eligible articles for further full text review. Based on a full text review, 100 articles were excluded for one or more of the following three reasons: 1) they do not address the physician staff, the adoption unit is either a nurse or administrative personnel; 2) the adoption unit is an organization, not physicians; and 3) they just describe critical adoption factors not supported by information and communication technology, for example, financial incentives to use electronic health records.

From all these studies we categorized the critical adoption factors into six main categories, which were arranged from the highest to the lowest order of relevance, as following user attitude towards information systems, workflow impact, interoperability, technical support, communication among users, and expert support. That order of importance is given by the frequency at which such factors are reported in the studies included in the review of literature. The 68 studies are listed in Table 2, where we show the nine aspects explained above for each study included. The critical adoption factors for adopting electronic health records for all included studies are summarized in Table 3 and described in turn.

**Table 2:** Critical factors for adopting clinical information systems included in the literature review

Study	Critical adoption factors	Country	Practice^a^	Setting^b^	Attention level^c^	Code for the critical adoption factor^d^	Use^e^	Study type^f^
Zheng, Padmana *et al. *[11]	Ease of use, time efficient, lack of relevance of the reminders, concerns about time and efficiency impacts, disruption in physician-patient communication.	USA	L	O	P	6	V	Q

Berner, Detmer *et al. *[6]	The development of scalable, interoperable systems. Communication among clinical systems.	USA	L	NS	NS	5	NS	D

Middleton, Hammond *et al. *[22]	Limited demonstrated value of electronic health records in practice, variability in the viability of the information systems, promotion of system standards.	USA	B	B	B	5, 6	NS	D

Poissant, Pereira *et al. *[15]	Time for patient care, user satisfaction, accuracy of the information, overall impact on workflow, the degree of exposure to a implemented system, reception of support from clinical leaders and training support, impact of system on an ensemble of work processes and outputs.	NS	NS	NS	NS	2, 3, 4, 6	NS	D

Poon, Jha *et al. *[27]	Lack of data standards, lack of interoperability between different data sources, negative impact of system implementation on productivity, usability issues, adaptability of the system to the different workflow patterns.	USA	B	B	B	2, 5, 6	NS	D

Liu, Wyatt *et al. *[34]	To bring about the intended user actions or behaviour.	NS	NS	NS	NS	6	NS	D

Ammenwerth, Iller et al. [24]	Usability and user friendliness of software, stability and flexibility of software, intensive user support, overall affects of the system on personnel workflow.	Germany	L	I	S	1, 2, 3, 4, 5, 6	NS	D

Sittig, Krall *et al. *[73]	Behind schedule alerts.	USA	L	B	B	2	M	C

Shah, Seger *et al. *[4]	Accurate clinical documentation, linkage of patient information from all clinical data repositories, minimized workflow interruptions.	USA	L	O	P	2, 4, 5, 6	M	Q

Ford, Menachemi *et al. *[2]	Influence and promote physicians' internal social networks.	USA	S	O	NS	1	NS	A

Chismar and Wiley-Patton [63]	Internet applications perceived usefulness, the importance and utility of the internet technology in performing daily tasks.	USA	S	NS	NS	6	V	Q

Simon, Kaushal et al. [64]	Loss of productivity.	USA	B	O	B	6	NS	Q

Despont-Gros, Mueller *et al. *[35]	Clinical information system characteristics (information system quality, interface characteristics, information quality), use/context/environment (ease of use, perceived usefulness), process characteristics (user participation).	NS	NS	NS	NS	6	NS	D

van der Meijden, Tange *et al. *[36]	System quality, information quality, user satisfaction, individual impact.	NS	NS	I	NS	6	NS	D

Ash and Bates [7]	Personal concerns about workflow, one-on-one communication and training.	USA	B	B	NS	1,2,3,4	NS	A

James [8]	Complexity of the systems and lack of data standards that permit exchange of clinical data, privacy concerns and legal barriers.	Europe, USA, Canada, Australia, New Zealand	S	O	P	5	NS	D

Callen, Braithwaite *et al. *[46]	Individual differences in terms of collaborative activities, authority level among physicians, and attitudes to -and use of- computers at the point of care.	Australia	L	I	S	1, 6	M	C

Chismar and Wiley-Patton [65]	Usefulness and job relevance.	USA	B	NS	S	6	V	Q

Hollingworth, Devine *et al. *[3]	Detrimental impact on workflow.	USA	L	O	S	2	M	C

Jerome, Giuse *et al. *[75]	Clinical workflow, newsletter services.	USA	L	O	P	1, 2	V	A

Joos, Chen *et al. *[25]	Communication among physicians, remote access, system speed, system efficiency, computer skill, computer-based documentation.	USA	L	O	P	1, 3, 4, 6	NS	D

Linder, Schnipper *et al. *[43]	Falling behind schedule, usability issues, concern about losing data, feeling that using the computer in front of the patient is rude.	USA	L	O	P	2, 6	M	C

Dixon and Stewart [44]	Ability and willingness to transfer knowledge and skills from one task to another, work knowledge.	Canada	NS	O	P	2, 6	V	C

Rouf, Chumley *et al. *[28]	Perform more complete histories and documentation; receive significantly more feedback from their preceptors on their electronic charts than on paper charts; concerns about the potential impact of the EHR on their ability to conduct the doctor-patient encounter.	USA	L	B	P	2, 4, 6	M	D

Saleem, Patterson *et al. *[26]	Integration of system to workflow, the ability to document system problems and receive prompt administrator feedback, poor usability.	USA	L	O	P	1, 2, 3, 6	M	D

Sequist, Cullen *et al. *[74]	Decreasing in the amount of time available to talk with patients, clinical productivity loss, available technical support.	USA	L	O	P	2, 3	M	C

Teich, Osheroff *et al. *[5]	Usability problems, lack of integration to important data from the system, uneven availability and management of best-practice system knowledge.	USA	L	O	P	3, 4, 5, 6	NS	D

Terry, Thorpe *et al. *[31]	The presence of a champion, training, the readiness of health care providers to accept the system.	Canada	L	O	P	3, 4, 6	NS	D

Zaidi, Marriott *et al. *[60]	System easy to learn, easy to show others how to use the system, easy to find additional information, and easy to use it within their daily workflow.	Australia	L	NS	NS	2, 6	M	Q

Krall and Sittig [47]	System ease of satisfying for work activities, and degree to which it support or disrupt workflow.	USA	L	O	P	2, 6	M	C

Krall and Sittig [29]	User centered design system, system perceived usefulness, alert or reminder must appear either at the appropriate time for consideration and action, or in a manner in which the user can determine if and when to evaluate and respond to it.	USA	L	O	P	2, 6	M	D

Aarts, Doorewaard *et al. *[53]	Compatibility of the system with the workflow, attitude of the users to information systems.	Germany	L	NS	NS	2, 6	M	A

Ash, Lyman *et al. *[45]	System usability, training, support, and time (compatibility with the workflow), communication among physicians.	USA	L	I	S	1, 2, 3, 4, 6	M	C

Audet, Doty *et al. *[79]	Lack of standard for information systems.	USA	B	NS	NS	5	NS	C

Bates, Cohen *et al. *[77]	Promote use of standards for data and systems; develop systems that communicate with each other.	USA	B	NS	NS	5	NS	D

Christensen and Grimsmo [48]	To find methods that can make a better representation of information in large patient records, prevent electronic patient records from contributing to increased administrative workload of physicians.	Norway	L	O	P	2, 6	V	C

Clayton, Narus *et al. *[52]	The perceived value of enhanced communications; the system functionality, response time and reliability; patient load of the physician in system learning phase.	USA	L	O	P	1, 2, 6	V	A

Gadd and Penrod [54]	Demonstration of value-added for the effort required to use electronic medical record, and its ability to facilitate efficient clinical workflows without negative effects.	USA	L	O	S	2, 6	NS	A

Granlien and Simonsen [78]	Poor integration with the general practitioners' existing IT systems.	Denmark	S	O	P	5	V	D

Halamka, Aranow *et al. *[33]	Interoperability limitations, lost productivity.	USA	L	B	B	5, 6	B	D

Kern, Barrón *et al. *[49]	Provide higher quality ambulatory care.	USA	S	O	P	6	V	C

Leung, Yu *et al. *[62]	Lack of technical support in case of system failure, lack of knowledge and perceived difficulty in learning new technology, lack of perceived benefits from computerization of clinical practice.	Hong Kong	NS	NS	NS	3, 6	NS	Q

Lo, Newmark *et al. *[55]	Time and workflow concerns.	USA	L	O	S	2, 6	V	A

Melles, Cooper *et al. *[37]	The flexibility of a computer interface, the speed and efficiency of a clinical computer system.	USA	L	O	S	6	NS	D

Menachemi, Ettel *et al. *[61]	The time needed to data entry in a system, the disruption of workflow, the lack of uniform data standards within the industry.	USA	B	B	B	2, 5, 6	NS	Q

Nilasena and Lincoln [58]	Focus on the end users' preferences in creating forms or screens to document care.	USA	L	O	S	6	V	E

Palm, Colombet *et al. *[57]	Overall service quality of the clinical information system.	France	L	I	S	6	NS	A

Pare, Sicotte *et al. *[50]	Psychological ownership of a clinical information system.	Canada	L	NS	NS	6	V	C

Payne, Perkins *et al. *[32]	Application functionality, speed, note writing time requirements, data availability, training need.	USA	L	I	S	3, 6	NS	D

Penrod and Gadd [51]	Improvements in quality and communications, impact on workflow.	USA	L	O	P	1, 2, 6	NS	A

Rodriguez, Murillo *et al. *[59]	Usability concerns in the graphical user interface of a system.	USA	L	NS	NS	6	NS	E

Rosenbloom, Grande *et al. *[30]	Integration of a system in the workflow, prefilled templates through simple typed entry, reuse captured notes on subsequent encounters with patients, interoperability of the system with other organization systems.	USA	L	I	S	2, 5, 6	V	D

Rosenbloom, Qi *et al. *[56]	Systems having greater functionality, workflow considerations.	USA	L	B	S	2, 6	V	A

Schade, Sullivan *et al. *[38]	Improved quality and consistency of care, practice efficiencies that have both timesaving and revenue generating effects, and potential shielding from malpractice claims.	UK	NS	O	P	6	NS	D

Vishwanath, and Scamurra [42]	Systems tend to not be very easy to use, loss of control over business processes, inability to master the system, lack of clear usefulness	USA	NS	NS	NS	6	NS	D

Stutman, Fineman *et al. *[39]	Frequency and utility of the alerts in a system.	USA	L	I	S	6	V	D

Tamblyn, Huang *et al. *[40]	Level to which the patient data are complex and fragmented.	Canada	S	O	P	6	V	D

Garrett, Brown *et al. *[41]	Usefulness and complexity of the system.	USA	B	B	NS	6	V	D

Weir, Lincoln *et al. *[76]	Early and intensive support, and 24 hour available assistance.	USA	L	B	B	3, 4	V	A

Lorenzi *et al. *[67]	Disturbs in workflow, electronics health records are more difficult to use than paper-based records.	USA	S	O	B	2, 6	NS	D

Morton and Wiedenbeck [86]	Provide technical support in a timely manner.	USA	L	NS	NS	3	NS	C

Rahimi *et al. *[68]	System was not adapted to their work routines; systems compatibility with professional values and needs, and its complexity of use.	Sweden	L	O	P	2, 6	M	C

Thyvalikakath *et al. *[70]	Problematic interface and interaction designs that led to usability problems.	USA	S	I	S	6	NS	C

Trivedi *et al. *[69]	Concerns about negative impact on workflow, potential need for duplication during the transition from paper to electronic systems of medical record keeping.	USA	L	O	S	2, 6	NS	C

Trimmer *et al. *[66]	The formal training and assistance by coworkers, the use of system knowledge base, the ease of use of the system.	USA	L	O	P	1, 3, 4, 6	M	D

DesRoches, Campbell *et al. *[87]	Quality of communication with other providers, timely access to medical records, avoiding medication errors, finding an electronic-records system to meet needs	USA	B	O	B	1, 6	NS	C

Bates [88]	System interoperability with other applications	USA	NS	NS	NS	5	NS	D

Kemper, Uren *et al. *[89]	No improvement in patient care or clinical outcomes, physician resistance, increase in physician workload, interference with doctor-patient relationship, inability to interface with existing systems	USA	B	O	P	5, 6	NS	C

^a ^Type of practice: S = small, L = large, B = Both, NS = Not specified

^b ^Type of setting: I = impatient, O = outpatient, B = Both, NS = Not specified

^c ^Attention level: P = Primary, S = Specialty, B = Both, NS = Not specified

^d ^Critical adoption factor: 1 = communication among users, 2 = workflow impact, 3 = technical support, 4 = expert support, 5 = interoperability, 6 = attitude towards information systems

^e ^System use: M = mandatory, V = voluntary, B = Both, NS = Not specified

^f ^Study type: D = descriptive; C = cross-sectional; A = Analytical (cross-sectional comparative, case control, cohort/prospective); E = Experimental; Q = Quasi-experimental (before-after, pre-experimental)

**Table 3:** Critical adoption factors for adopting clinical information systems in the reviewed studies

Critical factor	Number of studies by study type				
	
	Descriptive	Cross-sectional	Analytical	Experimental	Quasi-experimental
User attitude towards information systems	24 [5,15,22,24-42,66,67]	13 [43-50,68-70,87,89]	7 [51-57]	2 [58,59]	8 [4,11,60-65]

Workflow impact	8 [15,24,26-30,67]	10 [3,43-45,47,48,68,69,73,74]	8 [7,51-56,75]		3 [4,60,61]

Interoperability	11 [5,6,8,22,24,27,30,33,77,78,88]	2 [79,89]			2 [4,61]

Technical support	8 [5,15,24-26,31,32,66]	3 [45,74,86]	2 [7,76]		1 [62]

Communication among users	4 [24-26,66]	3 [45,46,87]	5 [2,7,51,52,75]		

Expert support	7 [5,15,24,25,28,31,66]	1 [45]	2 [7,76]		1 [4]

### User attitude towards information systems

We have defined user attitude towards information systems to characterize a subjective critical adoption factor of the electronic health records. As Ajzen [23] states, an attitude is a disposition to respond favorably or unfavorably to an object, person, institution, or event; it is a hypothetical construct that must be inferred from measurable responses. Attitude towards information systems is an important factor, as the information system behavior can be predicted based on this.

Fifty four studies determined user attitudes towards systems as a critical factor for adopting electronic health records [4,5,11,15,22,24-70,87,89]. Twenty four descriptive studies defined user attitude (interest, perceived usefulness, and motivation in working with it) towards these systems as a significant factor contributing to the user acceptance [5,15,22,24-42,66,67]. Thirteen cross-sectional studies determined that, to promote physicians' adoption of electronic health records, it is important to encourage and cultivate a positive attitude towards its use [43-50,68-70,87,89]. Seven analytical studies established the user attitude as a significant attribute associated with the electronic health records adoption [51-57]. Two experimental studies showed that the perceived characteristics of these systems' can significantly improve user acceptance and ease its adoption [58,59]. Eight quasi-experimental studies assessed physicians' attitude towards electronic health records and expressed preferences as a basis for system adoption [4,11,60-65]. Because user attitude towards information systems is a multivariate and subjective factor problem, it can be leveraged with information and communication technology roles focusing on modifying two important perceived characteristics of an innovation, namely: 1) perceived usefulness, which is defined as the extent to which individuals believe that using an information system would enhance their performance [71]; this may be supported with rapid access to knowledge within the organization. As a consequence, the adoption unit reduces the innovation uncertainty; and 2) perceived ease of use, explained as the degree to which an individual believes that using an information system will make his work easier [71]. This may be supported with friendly and context-aware user interfaces facilitating the realization of medical tasks. In the context of the innovation-decision process, these perceived characteristics are very important for all the five stages.

### Workflow impact

Workflow systems enable knowledge artifacts to be dispatched through organizations by means of a relative fixed process [72]. Twenty nine studies specify that clinical workflow must be taken into consideration to optimize the integration of electronic health records into the routine clinical practice [3,4,7,15,24,26-30,43-45,47,48,51-56,60,61,67-69,73-75]. Eight descriptive studies define that workflow has the potential to affect acceptance of these systems [15,24,26-30,67]. Ten cross-sectional surveys disclosed workflow as determinant for electronic health records acceptance [3,43-45,47,48,68,69,73,74]. Eight analytical studies define interference in workflow clinical activities as an impact factor for adopting the systems [7,51-56,75]. Three quasi-experimental studies argue that minimizing workflow interruptions supports high user acceptance in electronic health records [4,60,61]. Workflow knowledge artifacts can be embedded into electronic documents or into workflow information systems. Workflow electronic documents might be used to promote self learning, and assist users to reduce uncertainty about an innovation. In addition, workflow impact can modify the perceived characteristics of an innovation, which is critical in the persuasion stage of the innovation decision process.

### Interoperability

In this study, interoperability is related to compatibility, which is a perceived attribute of an innovation. The later is defined by Rogers [9] as the degree to which an innovation is perceived as being consistent with existent values, past experiences, and needs of a potential adoption unit. The term interoperability has been used because it is most related to electronic health records than compatibility. Fifteen studies determined interoperability as a determinant factor for adopting these systems [4-6,8,22,24,27,30,33,61,77-79,88,89]. Eleven descriptive studies established that interoperability could reduce rework by care providers, improve dissemination and movement of new medical knowledge among physicians [5,6,8,22,24,27,30,33,77,78,88]. Two cross-sectional study stated that interoperability is important because it decreases the cost of electronic health records and makes it feasible for an individual or small groups of physicians to acquire and adopt these systems [79,89]. Two quasi-experimental studies considered that non-interoperable electronic health records may negatively impact workflow and productivity, which in turn contributes to clinicians' resistance to adopt these systems [4,61]. Interoperability can be assisted with access to electronic documents about standards, and allowing electronic health records with access and compatibility to another information system. Interoperability might be important to determine the individual's behavior at the persuasion stage in the innovation-decision process.

### Technical support

Technical support relates to technical assistance given from technical staff to physicians through personal contact or via documents. Fourteen studies defined technical support as a critical adoption factor for electronic health records [5,7,15,24-26,31,32,45,62,66,74,76]. Eight descriptive qualitative studies specified that technical support must be given to physicians to promote the adoption of the systems [5,15,24-26,31,32,66]. Two cross-sectional surveys revealed technical support as determinant of electronic health records acceptance [45,74]. Two analytical studies established technical support as necessary, so that information of the use of the systems can be given at the time it is needed [7,76]. One quasi-experimental study defined the lack of technical support as a notable barrier to adopt electronic health records [62]. Both the social networks and the electronic technical documents, if they are given according to the user context, can encourage technical support. In the case of social networks, social interaction is promoted, whereas electronic technical documents aided self learning. Both social interactions and electronic technical artifacts might assist adoption [12]. In the decision stage of the innovation-decision process, technical support can cope with uncertainty about an innovation, and might be considered an important part of the resolution to adopt such innovation. Also, technical support can provide answers to the question "How do I use it?" in the innovation-decision process, which in turn can provide support to the implementation stage of the same. Finally, technical support also assists an adoption unit in the confirmation stage of the process for avoiding a reverse adoption decision.

### Communication among users

Communication among users refers to the act of interchanging thoughts, opinions, or information by speech, or writing. Twelve studies defined communication among users as a factor affecting the adoption of electronic health records [2,7,24-26,45,46,51,52,66,75,87]. Four descriptive studies defined communication among users as a very important factor contributing to the user acceptance of these systems [24-26,66]. Three cross-sectional studies showed that social networks are influential for change clinical behavior to diffuse electronic health records [45,46,87]. Five analytical studies showed that individuals must be provided with support for cooperation within a team for facilitating the adoption process of the systems [2,7,51,52,75]. It describes how, from a knowledge perspective, the communication among users might be encouraged through social networks to help innovation users promote social interaction, which assists them to adopt innovations. Based on Rogers [9], communication among users might be significant in the innovation-decision process for two reasons: 1) in the knowledge stage, it assists the characterization of the communication behavior of an adoption unit, and 2) in the confirmation stage, it helps the adoption unit to become aware of a need inducing it to look for information about an innovation to fulfill that need.

### Expert support

Expert support refers to the assistance provided from a physician to another physician. This can be divided in two aspects: 1) a physician with experience in electronic health records usage assists, with information about how to use the system, to another physician; 2) a physician has the knowledge to help another physician accomplish a medical task. Such assistance can be given through personal contact or via documents. Eleven studies established expert support as a very determinant factor for adopting electronic health records [4,5,7,15,24,25,28,31,45,66,76]. Seven descriptive studies recommended that centralized knowledge structures could reduce rework by physicians to facilitate the adoption of these systems [5,15,24,25,28,31,66]. One cross-sectional study claimed that expert support is important in spite of successful adoption of electronic health records [45]. Two analytical studies specified that expert support must be given to promote adoption of the systems [7,76]. One quasi-experimental study argued that expert support promotes high user acceptance in electronic health records [4]. Expert support might be provided to users in two forms: 1) enabling social networks to reduce uncertainly about these systems; and 2) through electronic knowledge artifacts to promote self learning. Because the social interaction and self learning help reduce uncertainly about innovations, both social networks and electronic knowledge artifacts may assist the adoption process. Like technical support, expert support deals with uncertainty concerning innovation, and in the context of innovation-decision process, three aspects might be considered: 1) providing the adoption unit with knowledge, fundamental part of adoption decision for the innovation, 2) supporting the implementation stage by helping adoption units to use an innovation, and 3) supporting the confirmation stage by providing supportive messages onto the adoption unit, and preventing an uncomfortable state of mind that may reverse an adoption decision.

### Limitations of the taxonomy

The main limitation of the taxonomy is the different impact of the adoption factors of electronic health records reported by some studies depending on the type of practice, setting, or attention level; however, these features are a determinant aspect with regard to the adoption rate for these systems rather than the presence of a specific critical adoption factor, as discussed in the next section.

## Discussion

In this section we present the main implications about the results of our literature review on critical adoption factors for adopting electronic health records. The following five aspects must be emphasized:

1) The definition of critical adoption factors described here does not make a difference between inpatient and outpatient settings for electronic health records. Nevertheless, as it has been explained in other studies, these differences represent variations in the level of the effect of a critical factor, rather than the presence of a specific critical adoption factor [7].

2) We have here depicted critical adoption factors for the use of electronic health records by physicians. But, it has been argued [7] that full advantage of these systems cannot be gained without intermediary staff, e.g. nursing personnel. However, we take into account only critical factors for adopting electronic health records by physicians, because to achieve adoption goals, these systems must be used by physicians, and this continues to be an important challenge [15,65].

3) Different kinds of practices present different kinds of adoption levels. For instance, small practices are less likely for people to adopt than large ones [80], and the adoption rate seems to be different between specialties and primary care [2]. However, these differences between practices just remark the adopter categories in the electronic health records adoption process and, therefore, this situation exclusively define the time it takes for a system to be adopted rather than deciding to adopt it. The definition of critical adoption factors defined here does not make a difference between types of practice.

4) Some studies indicate that national culture has a significant influence on a specific country adoption rates to some innovations [81]. On the other hand, different adoption rates for electronic health records in countries can be explained by national health services policies which make adoption of these systems more difficult and more expensive [82], although, as we stated before, these circumstances possibly define the only time it takes for a electronic health records to be adopted. However, the list of critical adoption factors specified here does not considered the country where studies were accomplished.

5) Our search does not differentiate between mandatory and voluntary settings for adoption studies. Adoption in voluntary use is reflected in the usage of the electronic health records, and for mandatory system use, the adoption is reflected in the overall user acceptance [24]. Therefore, a critical adoption factor taxonomy analyzed from a mandatory or voluntary setting perspective is a determinant aspect with regard to the adoption rate for electronic health records rather than causes affecting their adoption.

### Implications

**Figure 5:**
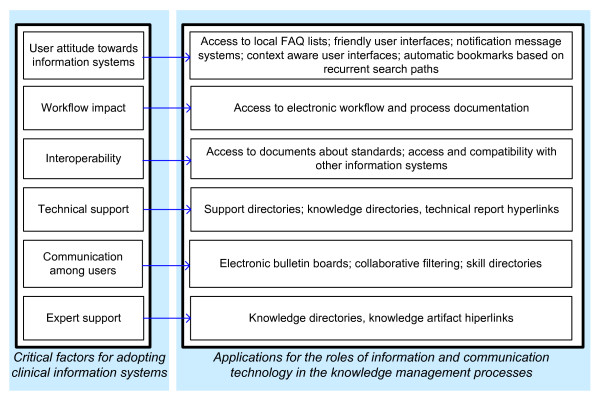
**Relationship among critical factors for adopting electronic health record systems with information and communication technology roles**.

Studies on identification of critical factors for adopting electronic health records show that it is possible to define a knowledge-oriented taxonomy for these factors (Figure 4). In this regard, our review provides a foundation for developing automatic support for assisting physicians in the process for adopting electronic health records. The critical adoption factors here defined propose the kind of knowledge that a user needs for the adoption process to be easier, these factors also help determine what kind of interaction occurs between them and the electronic health records, recommending the use of automatic mechanisms to support the adoption of those systems. Once identified, the information and communication technology roles in each knowledge management process can be related to the critical adoption factors of the electronic health records. The relationship between critical factors and the information and communication technology roles, shown in Figure 5, indicates which knowledge is necessary to reduce the uncertainty towards the electronic health records when a specific critical factor is considered. For example, the technical support has been reported as a critical adoption factor of the electronic health records [15]; if this factor is considered, an application of the technology roles of the knowledge management processes can be sought, and this application may support the communication amongst users. To provide this technical assistance in an easy and timely manner, a process for filtering information using techniques involving collaboration among multiple physicians could be useful.

The fact that some critical factors for adopting electronic health records were found to be dominant critical factors, may guide the efforts for assisting the adoption process of these systems. For example, attitude towards information systems and technical support are critical factors that may be supported with information and communication technology tools for facilitating knowledge dissemination, reducing uncertainty about the electronic health records. The former can be encouraged with friendly context-aware user interfaces which facilitate needed knowledge in the required format and timely; the later can be assisted by knowledge directories and hyperlinks to technical reports.

Taking into account a knowledge-based approach for defining critical adoption factors of electronic health records enables us, in addition, to define what critical adoption factor is determinant in each of the innovation-decision process stages. For example, as we have stated, communication among users is determinant in knowledge and confirmation stages, which can lead to use information and communication technology roles of knowledge management to moderate the communication behavior of an adoption unit, and help it become aware of an innovation to induce an adoption decision.

The information and communication technology roles described are available features in factual knowledge oriented information systems; and its relationship with the defined critical adoption factors can be used as a guide for a conceptual framework for assisting the adoption of electronic health records.

As we have stated, using a knowledge-based approach for classifying critical adoption factors of electronic health records leads to define a relationship between these factors and information and communication technology. These kinds of approaches imply transforming practical knowledge into structured one [83]. This is strongly related with the use of electronic artifacts. Then, considering that knowledge is a very influential factor in the innovation-decision process, and that the identified critical adoption factors are based on technology, the taxonomy here explained provides sound arguments on the importance and significance of assisting the latter process in two aspects: 1) transfer knowledge via electronic artifacts more effectively than via direct contact between information and communication technology users [84], and 2) the degree to which knowledge is structured corresponds to the extend to which knowledge is transferred [85].

Each specific type of interaction a physician has with an electronic health record could be associated with a critical adoption factor and the latter with a role of information and communication technology in the knowledge management processes; this association helps physicians to support an adoption decision in an automatic manner. Since it is possible to associate a physician- system interaction with the type of electronic health record adoption behavior, then it is possible to use the approach proposed in Figure 5 so that the critical factors are a guide to automatically improve the adoption of electronic health records, as explained below.

### User attitude towards information systems

User attitude towards electronic health records is a strong determinant for their adoption. Due to the subjective nature of this, there is not a specific type of interaction between the physician and the electronic health record that can make this attitude evident; however, it can be inferred through the system's use. For example, a transaction log of the system showing a slight interaction between physician and the electronic health record may indicate the perception of a negative attitude towards the system. If so, an electronic bulletin board where a physician can express his doubts, concerns, and general reasons for not using it can be used; from there the physician may be provided access to local frequent asked questions lists, or depending on the nature of the motive that led to his attitude, provide other information and communication technology tools to support an adoption decision.

### Workflow impact

Concerns about negative impact on workflow may cause uncertainty about the use of electronic health records, which can negatively impact on its adoption. For example, if a physician observes that the data filling a medical prescription form in the system reduces the time for patient care, the physician may stop using the features that this module provides. If the physician's interaction with the electronic health records indicates that he has stopped using that module, then it could automatically proposed prefilled templates through simple typed entry in this module, or reuse captured notes on subsequent encounters with patients.

### Interoperability

The lack of interoperability of the electronic health records with other electronic health records or organizational systems causes problems on their adoption. For example, if a physician needs to capture data in the system to provide health services in first level of attention and then reused the data in another unit system to provide other services, the physician requires that both systems are interoperable, if not, it can negatively affect his adoption decision. Although there is not a special classification of interactions between a physician and electronic health records that can indicate a lack of interoperability in a system, the adoption can be inferred based on their use. If this is the case, an electronic bulletin board can automatically provide assistance to understand the causes of non-use, when these are known then it can provide links to documents about standards or make reengineering of the system so that it can interact with other organizational units' systems or electronic health records.

### Technical support

The deficiency or lack of technical support makes the uncertainty about changes in electronic health records persisting, impacting negatively on its adoption. For example, if a physician performs the incomplete capture of data in a module of the system and does not receive technical support to investigate the reason and solve the problem, the physician may stop using module or the system. If the analysis of the interaction between the physician and the electronic health records reveals that the system did not capture the data in that module, the automatic provision of support directories or technical report hyperlinks to specific system information about a module can reduce uncertainty to capture data in this.

### Communication among users

There is a need of communication between the users of electronic health records, so that when an event that causes uncertainty about the use of the system occurs, coworkers can share their experience using the system, therefore promoting its adoption. For example, when modifying the electronic health record user interface, less experienced users can communicate with more experienced users so that the former can provide support to the latter in any doubt about the change. If the interaction between a physician and the system indicates that the characteristics associated with a change in the user interface was not used, then a tool that can relate users to help each other may be invoked automatically enabling the communication of the physician who did not used the user interface with experienced practitioners who could provide support to use it. Such strategies to support communication between physicians can be extended to other electronic health records users taking into account users' profiles: given that the tools for collaboration between users are based on their profiles, if these tools take into account a different profile, they can support different users.

### Expert support

When there is a lack or deficiency of medical expert support to other users of electronic health records that are less experienced, innovations may cause uncertainty about the use of the system, giving rise to problems in its adoption. For example, if a doctor has problems about a medical prescription in a module of the electronic health record and receives no assistance from his preceptor, the former could stop using the system. If the physician's interaction with the electronic health record indicates that the doctor has not made the prescription, the automatic provision of knowledge directories or knowledge artifact hyperlinks can provide the necessary knowledge to carry out the prescription.

## Conclusions

We have found that critical factors for adopting electronic health records can be classified from a knowledge oriented perspective to support the development of approaches for assisting the adoption of these systems.

As we stated before, the critical factors for adopting electronic health records to which we can provide automatic support by means of a knowledge perspective are six: user attitude towards information systems, workflow impact, interoperability, technical support, communication among users, and expert support. Based on the frequency with which such factors are reported in the studies included in the literature review, these are arranged from more prevailing to less prevailing factors.

Currently, there is little evidence of the use of information and communication technology to assist the process for adopting electronic health records in an automatic fashion. Focusing on the critical adoption factors here identified, benefits technology practitioners' efforts for designing and implementing information systems for supporting, in a proactive and knowledge oriented manner, the adoption of electronic health records. This provides four directions for future work: 1) defining knowledge representation structures for supporting the adoption of electronic health records; 2) scheming conceptual frameworks -and corresponding methods- for facilitating their adoption; 3) process-modeling for supporting in automatic manner the adoption of electronic health records; and 4) designing software architectures for software applications focused on supporting the adoption of factual electronic health records. We therefore consider that critical adoption factors and its relationship with the information and communication technology roles of the knowledge management processes provide sound theoretical basis that scholars should use for empirical and theoretical research to understand, support, and facilitate the physician's adoption of information and communication technology for the benefit of patients.

## Competing interests

The authors declare that they have no competing interests.

## Authors' contributions

VHC and AIMG developed the idea for the study and its design. All authors reviewed the literature. VHC and JRGP drafted the manuscript. All authors revised the manuscript critically and approved its final version.

## Pre-publication history

The pre-publication history for this paper can be accessed here:

http://www.biomedcentral.com/1472-6947/10/60/prepub
